# Supplementary material for: MetaRibo-Seq measures translation in microbiomes
Source: Nat Commun. 2020 Jun 29;11:3268. doi: 10.1038/s41467-020-17081-z (PMC7324362; doi:10.1038/s41467-020-17081-z)
Supplement: Supplementary file 10 — Supplementary Data 7 [file 41467_2020_17081_MOESM10_ESM.zip › File2/Confidence_High_Taxonomy/290658_out.krona.html]

Javascript must be enabled to view this page.

members
magnitude
magnitudeUnassigned
count
unassigned
taxon
rank

290658\_out

444

2
superkingdom
444

phylum
443
976

class
443
200643

order
443
171549

family
443
815

genus

SRS013098\_contig\_number\_contig-100\_1861.297354SRS013800\_contig\_number\_4656SRS014235\_contig\_number\_35233SRS015065\_contig\_number\_3934SRS017191\_contig\_number\_834SRS017433\_contig\_number\_11264SRS019685\_contig\_number\_38505SRS020271\_contig\_number\_contig-100\_3761.3762SRS020869\_contig\_number\_12843SRS021948\_contig\_number\_7864SRS024625\_contig\_number\_980SRS049995\_contig\_number\_1610SRS052027\_contig\_number\_17066SRS055982\_contig\_number\_18696SRS057478\_contig\_number\_5912SRS063040\_contig\_number\_10418SRS077641\_contig\_number\_contig-100\_1711.131787SRS1055076\_contig\_number\_contig-100\_241.60525SRS140645\_contig\_number\_1262SRS147139\_contig\_number\_contig-100\_2999.118939SRS147346\_contig\_number\_42585SRS148511\_contig\_number\_16584SRS893230\_contig\_number\_7442SRS893300\_contig\_number\_8933
443
24
816

817

SRS018817\_contig\_number\_9778SRS024549\_contig\_number\_4196SRS043001\_contig\_number\_796SRS098655\_contig\_number\_1925SRS143598\_contig\_number\_5817
5
species

1
species
291644

SRS149325\_contig\_number\_11271


SRS019030\_contig\_number\_4534SRS054352\_contig\_number\_259SRS078176\_contig\_number\_3579SRS146764\_contig\_number\_1039SRS893369\_contig\_number\_8609
2292941
species
5


SRS023715\_contig\_number\_26058
47678
species
1

329854

SRS018623\_contig\_number\_2478SRS148319\_contig\_number\_5336
2
species

310298

SRS042628\_contig\_number\_33149
1
species

1
species
28111

SRS1055099\_contig\_number\_13615

246787

SRS016203\_contig\_number\_2098SRS075984\_contig\_number\_20419SRS078419\_contig\_number\_13281SRS140513\_contig\_number\_874
4
species

species
1

SRS015217\_contig\_number\_17264
820


SRS019445\_contig\_number\_16157
338188
species
1

species
1

SRS013521\_contig\_number\_9053
371601

species
16

SRS018049\_contig\_number\_352SRS018541\_contig\_number\_2442SRS019286\_contig\_number\_6072SRS024132\_contig\_number\_14082SRS024435\_contig\_number\_3636SRS052078\_contig\_number\_3SRS054590\_contig\_number\_113SRS057717\_contig\_number\_513SRS063985\_contig\_number\_27360SRS075341\_contig\_number\_15203SRS077024\_contig\_number\_10759SRS101268\_contig\_number\_17SRS104636\_contig\_number\_16664SRS1055034\_contig\_number\_5099SRS1055067\_contig\_number\_1441SRS142712\_contig\_number\_3123
357276


SRS011084\_contig\_number\_37411SRS011239\_contig\_number\_23781SRS011302\_contig\_number\_22228SRS011405\_contig\_number\_2396SRS012902\_contig\_number\_230SRS012969\_contig\_number\_3551SRS013158\_contig\_number\_1569SRS013215\_contig\_number\_46SRS013382\_contig\_number\_2379SRS013638\_contig\_number\_15019SRS013687\_contig\_number\_32588SRS013940\_contig\_number\_4655SRS013965\_contig\_number\_28493SRS014287\_contig\_number\_16071SRS014313\_contig\_number\_4209SRS014412\_contig\_number\_1126SRS014534\_contig\_number\_51SRS014736\_contig\_number\_7063SRS014855\_contig\_number\_1098SRS014948\_contig\_number\_16984SRS014979\_contig\_number\_17900SRS015190\_contig\_number\_3376SRS015264\_contig\_number\_16699SRS015431\_contig\_number\_76672SRS015578\_contig\_number\_7932SRS015579\_contig\_number\_17238SRS015663\_contig\_number\_4475SRS015794\_contig\_number\_7777SRS015854\_contig\_number\_2297SRS015890\_contig\_number\_13045SRS015960\_contig\_number\_16907SRS016018\_contig\_number\_10669SRS016095\_contig\_number\_11510SRS016132\_contig\_number\_1736SRS016335\_contig\_number\_18372SRS016381\_contig\_number\_2492SRS016495\_contig\_number\_15509SRS016753\_contig\_number\_contig-100\_803.47518SRS016840\_contig\_number\_2244SRS016897\_contig\_number\_625SRS016989\_contig\_number\_11404SRS017103\_contig\_number\_6225SRS017247\_contig\_number\_393SRS017307\_contig\_number\_13673SRS017375\_contig\_number\_856SRS017521\_contig\_number\_3205SRS017701\_contig\_number\_19567SRS017745\_contig\_number\_contig-100\_192.35630SRS017859\_contig\_number\_2719SRS018351\_contig\_number\_5744SRS018575\_contig\_number\_4986SRS018836\_contig\_number\_14080SRS018888\_contig\_number\_contig-100\_306.56691SRS018936\_contig\_number\_9304SRS018984\_contig\_number\_502SRS019068\_contig\_number\_7790SRS019178\_contig\_number\_3834SRS019267\_contig\_number\_1475SRS019397\_contig\_number\_12497SRS019496\_contig\_number\_10680SRS019601\_contig\_number\_20602SRS019693\_contig\_number\_1391SRS019808\_contig\_number\_11576SRS019910\_contig\_number\_24963SRS019968\_contig\_number\_11725SRS020394\_contig\_number\_14255SRS020508\_contig\_number\_17SRS020622\_contig\_number\_770SRS021153\_contig\_number\_1490SRS021219\_contig\_number\_4086SRS021484\_contig\_number\_31882SRS022071\_contig\_number\_22321SRS022137\_contig\_number\_17270SRS022524\_contig\_number\_15944SRS022713\_contig\_number\_1687SRS023526\_contig\_number\_17092SRS023583\_contig\_number\_5643SRS023829\_contig\_number\_1003SRS023914\_contig\_number\_3520SRS023971\_contig\_number\_1589SRS024009\_contig\_number\_7345SRS024331\_contig\_number\_20059SRS024492\_contig\_number\_10473SRS024663\_contig\_number\_9036SRS042284\_contig\_number\_108SRS043411\_contig\_number\_3043SRS043667\_contig\_number\_2248SRS043701\_contig\_number\_11734SRS043841\_contig\_number\_8977SRS045244\_contig\_number\_448SRS045645\_contig\_number\_29935SRS045713\_contig\_number\_11071SRS045739\_contig\_number\_1708SRS045826\_contig\_number\_12350SRS046068\_contig\_number\_495SRS046369\_contig\_number\_4224SRS046502\_contig\_number\_13662SRS046717\_contig\_number\_10461SRS046810\_contig\_number\_9SRS047014\_contig\_number\_34604SRS047044\_contig\_number\_12571SRS047433\_contig\_number\_11550SRS048164\_contig\_number\_25269SRS048870\_contig\_number\_10508SRS048981\_contig\_number\_94SRS049402\_contig\_number\_8735SRS049712\_contig\_number\_14859SRS049773\_contig\_number\_9125SRS049896\_contig\_number\_19838SRS049959\_contig\_number\_2680SRS050299\_contig\_number\_838SRS050422\_contig\_number\_19506SRS050520\_contig\_number\_358SRS050752\_contig\_number\_16648SRS050974\_contig\_number\_contig-100\_68.24107SRS050998\_contig\_number\_15536SRS051031\_contig\_number\_37241SRS051610\_contig\_number\_923SRS051882\_contig\_number\_9836SRS052576\_contig\_number\_289SRS052697\_contig\_number\_13881SRS053087\_contig\_number\_contig-100\_111.44557SRS053214\_contig\_number\_1469SRS053335\_contig\_number\_17337SRS053356\_contig\_number\_47495SRS053398\_contig\_number\_2839SRS053649\_contig\_number\_17339SRS054059\_contig\_number\_4508SRS054905\_contig\_number\_3964SRS055017\_contig\_number\_15294SRS055966\_contig\_number\_12SRS056259\_contig\_number\_12043SRS056273\_contig\_number\_3380SRS056519\_contig\_number\_3096SRS056537\_contig\_number\_39590SRS057480\_contig\_number\_contig-100\_253.75096SRS058070\_contig\_number\_24976SRS058145\_contig\_number\_contig-100\_96.97SRS058268\_contig\_number\_18SRS058723\_contig\_number\_220SRS062427\_contig\_number\_151SRS062701\_contig\_number\_4769SRS063127\_contig\_number\_910SRS063190\_contig\_number\_11200SRS063370\_contig\_number\_8722SRS063489\_contig\_number\_4492SRS063518\_contig\_number\_contig-100\_2875.206142SRS063813\_contig\_number\_5929SRS063928\_contig\_number\_contig-100\_656.13492SRS064232\_contig\_number\_1097SRS064757\_contig\_number\_18878SRS064973\_contig\_number\_85SRS065176\_contig\_number\_7943SRS065397\_contig\_number\_3372SRS065504\_contig\_number\_36265SRS074586\_contig\_number\_4581SRS074670\_contig\_number\_924SRS074964\_contig\_number\_2609SRS075021\_contig\_number\_contig-100\_463.53711SRS075078\_contig\_number\_21138SRS075398\_contig\_number\_306SRS075716\_contig\_number\_4019SRS075773\_contig\_number\_30628SRS075821\_contig\_number\_15837SRS075878\_contig\_number\_5146SRS076756\_contig\_number\_10035SRS076876\_contig\_number\_85SRS077086\_contig\_number\_2402SRS077127\_contig\_number\_13161SRS077194\_contig\_number\_10849SRS077231\_contig\_number\_16236SRS077335\_contig\_number\_3350SRS077392\_contig\_number\_contig-100\_1132.252579SRS077454\_contig\_number\_9718SRS077502\_contig\_number\_3001SRS077589\_contig\_number\_1442SRS077730\_contig\_number\_12549SRS077849\_contig\_number\_20206SRS077991\_contig\_number\_contig-100\_13.14SRS078242\_contig\_number\_16398SRS097889\_contig\_number\_34220SRS097920\_contig\_number\_6658SRS097958\_contig\_number\_1721SRS098073\_contig\_number\_17897SRS098571\_contig\_number\_7343SRS098644\_contig\_number\_24689SRS098717\_contig\_number\_13201SRS098827\_contig\_number\_3026SRS100907\_contig\_number\_contig-100\_23.62465SRS101376\_contig\_number\_25977SRS101433\_contig\_number\_7712SRS103987\_contig\_number\_395SRS104036\_contig\_number\_4200SRS104084\_contig\_number\_1860SRS1041031\_contig\_number\_26560SRS1041032\_contig\_number\_4205SRS1041036\_contig\_number\_2150SRS1041037\_contig\_number\_9562SRS1041038\_contig\_number\_14529SRS1041039\_contig\_number\_19439SRS1041090\_contig\_number\_5191SRS1041092\_contig\_number\_8466SRS1041112\_contig\_number\_2826SRS1041116\_contig\_number\_7719SRS1041118\_contig\_number\_10243SRS1041129\_contig\_number\_10735SRS1041130\_contig\_number\_85SRS1041133\_contig\_number\_25225SRS1041134\_contig\_number\_2185SRS1041136\_contig\_number\_10688SRS1041137\_contig\_number\_15162SRS1041138\_contig\_number\_9406SRS1041139\_contig\_number\_1126SRS1041140\_contig\_number\_18403SRS1041141\_contig\_number\_891SRS1041143\_contig\_number\_6471SRS1041144\_contig\_number\_8607SRS1041145\_contig\_number\_31888SRS1041147\_contig\_number\_1101SRS1041157\_contig\_number\_5336SRS1041164\_contig\_number\_200SRS104327\_contig\_number\_1921SRS104400\_contig\_number\_13425SRS104485\_contig\_number\_52SRS104912\_contig\_number\_20124SRS104975\_contig\_number\_17112SRS105082\_contig\_number\_10195SRS105153\_contig\_number\_25396SRS1054716\_contig\_number\_9959SRS1054928\_contig\_number\_5127SRS1055022\_contig\_number\_14235SRS1055043\_contig\_number\_9857SRS1055049\_contig\_number\_1080SRS1055056\_contig\_number\_9025SRS1055069\_contig\_number\_4SRS1055083\_contig\_number\_371SRS1055095\_contig\_number\_365SRS140492\_contig\_number\_11537SRS142503\_contig\_number\_29103SRS142542\_contig\_number\_5232SRS142599\_contig\_number\_29949SRS142781\_contig\_number\_186SRS142890\_contig\_number\_21962SRS142923\_contig\_number\_22134SRS142980\_contig\_number\_5517SRS143085\_contig\_number\_1405SRS143148\_contig\_number\_24467SRS143181\_contig\_number\_12104SRS143342\_contig\_number\_10149SRS143372\_contig\_number\_8745SRS143417\_contig\_number\_28521SRS143722\_contig\_number\_9680SRS143780\_contig\_number\_5068SRS143876\_contig\_number\_1292SRS143895\_contig\_number\_44434SRS144135\_contig\_number\_33095SRS144183\_contig\_number\_38716SRS144297\_contig\_number\_2027SRS144362\_contig\_number\_33629SRS144506\_contig\_number\_21197SRS144537\_contig\_number\_47949SRS144603\_contig\_number\_12292SRS144714\_contig\_number\_2818SRS144753\_contig\_number\_9795SRS145308\_contig\_number\_227SRS145497\_contig\_number\_15803SRS146812\_contig\_number\_49947SRS146813\_contig\_number\_2932SRS147022\_contig\_number\_265SRS147039\_contig\_number\_4775SRS147271\_contig\_number\_1241SRS147377\_contig\_number\_269SRS147425\_contig\_number\_4622SRS147557\_contig\_number\_34132SRS147614\_contig\_number\_7075SRS147653\_contig\_number\_5800SRS147766\_contig\_number\_17438SRS147977\_contig\_number\_183SRS148159\_contig\_number\_41172SRS148196\_contig\_number\_34615SRS148253\_contig\_number\_6681SRS148424\_contig\_number\_27114SRS148721\_contig\_number\_36227SRS148784\_contig\_number\_25465SRS148817\_contig\_number\_1215SRS148970\_contig\_number\_603SRS149075\_contig\_number\_7265SRS149181\_contig\_number\_30839SRS149244\_contig\_number\_4359SRS149784\_contig\_number\_11719SRS149879\_contig\_number\_21310SRS892882\_contig\_number\_12983SRS893170\_contig\_number\_1288SRS893187\_contig\_number\_17698SRS893252\_contig\_number\_10810SRS893253\_contig\_number\_260SRS893256\_contig\_number\_51SRS893270\_contig\_number\_19587SRS893279\_contig\_number\_10615SRS893285\_contig\_number\_138SRS893288\_contig\_number\_302SRS893292\_contig\_number\_1789SRS893295\_contig\_number\_1882SRS893327\_contig\_number\_2447SRS893342\_contig\_number\_8539SRS893358\_contig\_number\_9656SRS893373\_contig\_number\_20426SRS893378\_contig\_number\_18369SRS893382\_contig\_number\_5461SRS893383\_contig\_number\_22677SRS971275\_contig\_number\_39828
821
species
311

2
species
28116

SRS013476\_contig\_number\_36996SRS098061\_contig\_number\_7967

species
3

SRS014613\_contig\_number\_14534SRS064276\_contig\_number\_2892SRS1054691\_contig\_number\_11247
2292935

1
species
376806

SRS048060\_contig\_number\_1902

species
4

SRS020233\_contig\_number\_23717SRS1041091\_contig\_number\_17699SRS147088\_contig\_number\_4499SRS893172\_contig\_number\_9273
204516

2292939

SRS016629\_contig\_number\_3961SRS022609\_contig\_number\_19223SRS1041095\_contig\_number\_contig-100\_3669.27675
3
species

1
species
387090

SRS019381\_contig\_number\_16355


SRS148091\_contig\_number\_12554
469590
species
1

species
2

SRS024388\_contig\_number\_1882SRS1041132\_contig\_number\_521
671267

species
1

SRS011134\_contig\_number\_16406
626930


SRS014459\_contig\_number\_5192SRS015486\_contig\_number\_761SRS016056\_contig\_number\_244SRS016954\_contig\_number\_5387SRS018133\_contig\_number\_2840SRS018427\_contig\_number\_22146SRS020328\_contig\_number\_15221SRS042966\_contig\_number\_19946SRS043768\_contig\_number\_24425SRS045195\_contig\_number\_6572SRS049446\_contig\_number\_7978SRS049900\_contig\_number\_22SRS050801\_contig\_number\_3871SRS050941\_contig\_number\_11373SRS064557\_contig\_number\_11294SRS064645\_contig\_number\_64SRS076976\_contig\_number\_21101SRS077294\_contig\_number\_11873SRS077552\_contig\_number\_1976SRS078665\_contig\_number\_1368SRS100021\_contig\_number\_2759SRS1041142\_contig\_number\_4464SRS1055038\_contig\_number\_1072SRS143991\_contig\_number\_1236SRS148874\_contig\_number\_5625SRS150029\_contig\_number\_11482SRS893341\_contig\_number\_1287
2292944
species
27

1262754

SRS013951\_contig\_number\_4326SRS019161\_contig\_number\_19426
2
species

4
species
818

SRS045004\_contig\_number\_7408SRS104693\_contig\_number\_162SRS147139\_contig\_number\_20977SRS893366\_contig\_number\_20406

2292936

SRS143523\_contig\_number\_567
1
species

species
1

SRS143070\_contig\_number\_15865
310297

species
1

SRS015369\_contig\_number\_369
1638779


SRS012273\_contig\_number\_44395SRS014683\_contig\_number\_5369SRS015694\_contig\_number\_4612SRS024075\_contig\_number\_9540SRS044535\_contig\_number\_18359SRS050026\_contig\_number\_2681SRS050925\_contig\_number\_299SRS058770\_contig\_number\_1813SRS062654\_contig\_number\_14499SRS098881\_contig\_number\_15075SRS893259\_contig\_number\_1345SRS893380\_contig\_number\_59SRS971276\_contig\_number\_28484
46506
species
13

species
2

SRS014923\_contig\_number\_contig-100\_870.247347SRS023346\_contig\_number\_4358
291645

1
phylum
1239

91061
class
1

186826
1
order

family
1
186828

genus
1
2747


SRS015782\_contig\_number\_1943
1637504
species
1
